# A 74-year-old soybean allergy woman with anaphylaxis after propofol injection

**DOI:** 10.1186/2045-7022-3-S3-P147

**Published:** 2013-07-25

**Authors:** AS Jang, C-S Park, DJ Kim, S-W Park

**Affiliations:** 1 Soonchunhyang University Bucheon Hospital, Bucheon, Republic of Korea

## Background

Adverse immune responses to foods affect approximately 3% to 4% of adults in westernized countries and appear to have increased in prevalence. Food induced allergic reactions are responsible for a variety of symptoms and disorders involving the skin and gastrointestinal and respiratory tracts and can be attributed to IgE-mediated and non–-IgE-mediated (cellular) mechanisms.

## Methods

A previously healthy 74-year-old woman visited our hospital to undergo esophagogastroduodenoscopy for a check-up. She had an allergy to soybean. Approximately 1 min following administration of intravenous propofol, stridor was heard and oxygen saturation fell to 56% on pulse oximetry. We decided to stop the exam, and tried to remove the endoscope. But, the endoscope has stuck in her throat and it was not pulled out. We removed the endoscope by compulsion. After 10 seconds endoscope removal, severe wheezing was heard and her oxygen saturation fell to 56%. The larynx was observed using a laryngoscope for endotracheal intubation. At that time marked swelling and severe edema of the epiglottis and heavy mucoid secretion in the epiglottis extending to the arytenoids cartilage was detected. Immediately 1mg epinephrine was administered subcutaneously, together with 150 mg intravenous methylprednisolone was infused. Because the patient was not improved in symptom after 1 minute, 1mg epinephrine was administered intravenously. After 1minute, her oxygen saturation recovered to 98% and the wheezing was subsided.

## Results

The patient underwent skin-prick testing 14 days after the event. Skin-prick tests with dilutions of propofol and 20% Intralipid (Smoflipid, Fresenius Kabi, Bad Homburg, Germany), and phenol showed immediate reaction for propofol and 20% Intralipid.

## Conclusion

So we suspected soybean in intralipid, a component of propofol as the cause of this anaphylaxis, because she had a food allergy history to soybean and she showed positive responses to intralipid and propofol on skin-prick testing. She was informed of the results and of the risk for anaphylaxis if re-exposed to propofol or nutritional supplements containing soybean in the future.

## Disclosure of interest

None declared.

